# Multiple idiopathic external cervical root resorption in patient treated continuously with denosumab: a case report

**DOI:** 10.1186/s12903-022-02165-7

**Published:** 2022-04-15

**Authors:** Katarína Mikušková, Peter Vaňuga, Katarína Adamicová, Dagmar Statelová, Mária Janíčková, Igor Malachovský, Tomáš Siebert

**Affiliations:** 1 Department of Stomatology and Maxillofacial Surgery, Jessenius Faculty of Medicine in Martin, University Hospital, Martin, Comenius University in Bratislava, Kollárova 2, 036 01 Martin, Slovakia; 2 National Institute of Endocrinology and Diabetology, Kollárova 282/3, 034 91 Lubochna, Slovakia; 3 Department of Pathology, Jessenius Faculty of Medicine in Martin, University Hospital, Martin, Comenius University in Bratislava, Kollárova 2, 036 01 Martin, Slovakia

**Keywords:** External cervical root resorption, Denosumab, Bone turnover, Osteoporosis, Antiresorptive therapy

## Abstract

**Background:**

External root resorption is an irreversible loss of dental hard tissue as a result of odontoclastic action. Multiple external cervical root resorptions in permanent teeth are rare. The exact cause of external cervical root resorption is unclear. It is currently well established that RANK/RANKL signaling is essential for osteoclastogenesis and osteoclast-mediated bone resorption. Denosumab is an anti-RANKL antibody used for the treatment of postmenopausal osteoporosis. RANK/RANKL pathway suppression by denosumab is expected to suppress the activity of clastic cells responsible for hard tissue resorption involving both osteoclasts and odontoclasts.

**Case presentation:**

This case report demonstrates aggressive and generalized idiopathic external cervical root resorption that started and advanced during ongoing antiresorptive therapy with the human monoclonal RANKL-blocking antibody denosumab without discontinuation of therapy in a 74-year-old female patient treated for postmenopausal osteoporosis. The extent of resorptive defects was too large and progressively led to fractures of the teeth. The number of teeth involved and the extend of destruction excluded conservative treatment. The affected teeth had to be extracted for functional prosthetic reconstruction.

**Conclusions:**

This finding suggests that treatment with denosumab may be associated with severe and aggressive odontoclastic resorption of multiple dental roots despite an adequate inhibitory effect on osteoclasts in the treatment of osteoporosis. The RANKL-independent pathways of clastic cell formation are likely to be involved in this pathological process.

## Background

External cervical root resorption (ECR) is an irreversible loss of hard dental structures that may necessitate dental treatment or even an extraction of affected teeth. Multiple external resorptions in permanent dentition are uncommonly reported phenomena. The etiology and pathogenesis of ECR is not exactly understood, but it is believed to involve the action of osteoclast-like cells (odontoclasts) originating from hematopoietic stem cells in the bone marrow [1]. In addition, it has been suggested that the presence of inflamed fibrovascular connective tissue adjacent to the root surface lacking an intact periodontal ligament is a prerequisite for cervical root resorption [1]. Although several cases of ECR have been reported as idiopathic, various mechanical or chemical factors have been associated with ECR, particularly dental trauma and orthodontic treatment [2, 3]. Most cases of ECR are asymptomatic and are typically discovered as an incidental finding on radiographic examination [4]. The pathologic resorptive process typically starts below epithelial attachment at the mesial or distal cemento-enamel junction of the tooth and can progress and involve the entire cervical region [5]. ECR is a result of increased odontoclastic activity that develops as a consequence of local damage or deficiency of the periodontal ligament or subepithelial cementum [3]. *R*eceptor activator of nuclear factor kappaB (RANK) is a *receptor* for *RANK* ligand (RANKL) and part of the *RANK*/RANKL/osteoprotegrin (OPG) signaling pathway that regulates osteoclast differentiation and activation. RANK/RANKL/OPG plays an essential role in osteoclastogenesis and osteoclastic bone resorption [6]. Histological studies show that local trauma to the bone or periodontal ligament increases the concentrations of RANKL, macrophage-colony stimulating factor (M-CSF), tumor necrosis factor alpha (TNF-α), interleukin 1β (IL-1β) and other inflammatory cytokines that stimulate osteoclast and odontoclast differentiation [7–9]. Denosumab is a novel variant of antiresorptive therapy used to treat osteoporosis (e.g., Prolia 60 mg every 6 months) or to treat or prevent skeletal complications in malignancies (e.g., Xgeva 120 mg up to once every month) [10]. Denosumab acts as a human monoclonal antibody that prevents the RANK/RANKL interaction, thereby inhibiting osteoclast development and activation [11]. The long-term efficacy and safety of denosumab has been evaluated in the FREEDOM Extension Trial with results published for up to 10 years of denosumab exposure, demonstrating a continuing increase in bone mineral density, a sustained reduction in bone turnover markers, a low fracture incidence and a consistent safety profile [12]. Antiresorptive therapy with denosumab inhibits RANK/RANKL signaling and thereby osteoclast-mediated bone resorption, suggesting the same effect for odontoclast-mediated root resorption [13, 14]. The prevalence rate of ECR in the general population varies from 0.02 to 0.08% [15]. Evidence on the prevalence rate of ECR in individuals affected by osteoporosis or treated with denosumab is lacking.

In the present article, we demonstrate the coincidence of long-term and continuous antiresorptive therapy with denosumab and aggressive multiple external cervical root resorptions in patient with postmenopausal osteoporosis.

## Case presentation

**Fig. 1:**
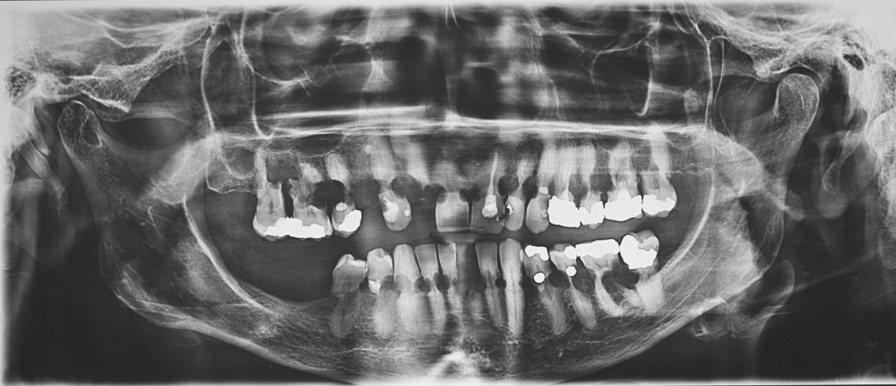
A panoramic radiograph taken in July 2020 showing generalized external cervical root resorption

**Fig. 2:**
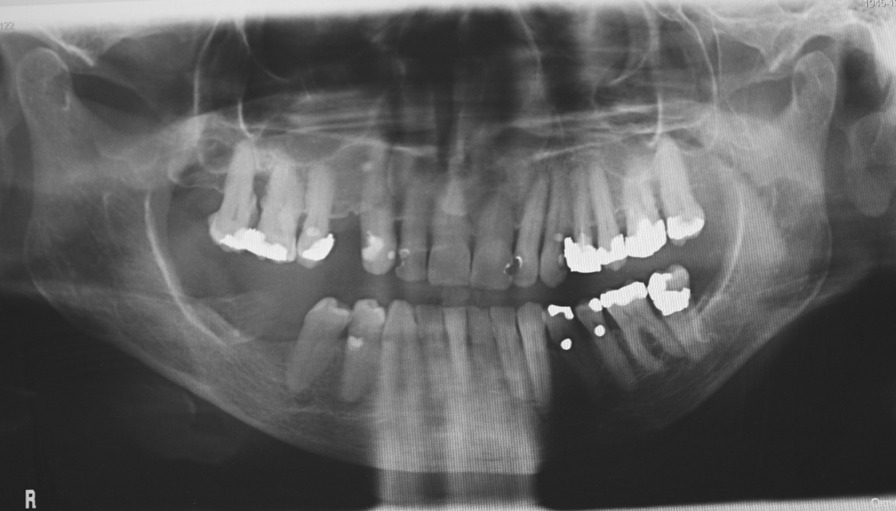
A panoramic radiograph taken in September 2017 with no signs of external cervical root resorption

A 74-year-old female patient presented in July 2020 with a chief complaint of occasional pain, thermal sensitivity and slightly increased mobility of her left mandibular second premolar (tooth 35). Her symptoms started two months ago. She also reported unexpected fractures of otherwise asymptomatic teeth in the previous weeks. Four weeks prior to the first examination, a crown of the first left mandibular incisor (tooth 31) suddenly broke. Two weeks later, in a similar manner, she lost a crown of the second maxillary right incisor (tooth 12) because of the fracture in the cervical area of the tooth caused by chewing. She was referred by her dentist to the Department of Stomatology and Maxillofacial Surgery at University Hospital Martin with numerous sites of external cervical root resorption found on her recent panoramic radiograph (Fig. 1). All roots of her 23 teeth were affected, and crowns of previously broken teeth 12 and 31 were missing. There were no signs of external cervical root resorption on her previous radiographic examination in 2017. However, the panoramic radiograph from 2017 showed signs of Generalized Stage 3 Periodontitis with moderate to severe bone loss around most maxillary and several mandibular teeth. Subgingival calculus was noted on maxillary molars. Bone loss around these teeth was severe and consistent with presence of calculus. Significant bone loss was present around maxillary premolars, between incisors, and between maxillary right canine and lateral incisor. (Fig. 2).

There was no history of dental trauma, periodontal surgery, intracoronal bleaching or Paget disease of bone. Her medical history was positive for arterial hypertension, glaucoma and postmenopausal osteoporosis with multiple osteoporotic vertebral fractures in the thoracic spine. Her treatment for osteoporosis started in 2008 with parathormone injections. Because of an allergic reaction to parathormone, the therapy was changed to strontium renalete for the next two years. Since 2011, she has been administered antiresorptive therapy with the human monoclonal antibody denosumab (60 mg) via subcutaneous injections every six months without any discontinuation until the first presentation to our department. She received the last injection of denosumab in March 2020. At the time of her presentation to our clinic, she was medicated with the following medications: denosumab, calcium, vitamin D, nitredipine, perindopril, bisoprolol, latanoprost, and timolol.

**Fig. 3:**
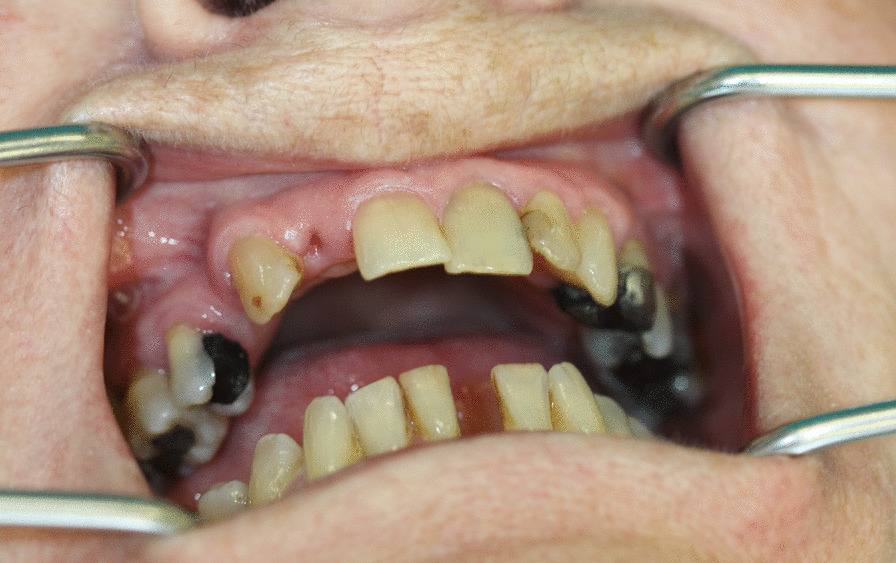
Intraoral photograph taken in July 2020. Missing crowns of recently broken teeth 12, 31

No detectable abnormalities were identified on extraoral and intraoral examination. Intraoral examination revealed good oral hygiene, dentition with multiple restorations and no active carious lesions. Crowns of previously broken teeth 31 and 12 were missing, and the roots were covered with gingiva (Fig. 3). Tooth 35 showed slightly increased mobility, and all other teeth were not discolored and did not exhibit increased mobility. Despite the radiographically noticeable extensive loss of root structure, all teeth tested vital with the exception of earlier endodontically treated teeth 21 and 24 and molars 26 and 37 with massive metallic restorations. Her current panoramic radiograph showed aggressive external cervical root resorption in all 23 teeth. Most resorptive lesions started in the approximal cervical area of the root and spread towards the pulp or towards the apex of the root. The resorptive process extended beyond the coronal third of the root, particularly for teeth 17, 27, and 36. There was also a resorptive defect in the apical area of tooth 16. Attempted probing of the periodontal sulcus of the upper central incisors with a periodontal probe failed to penetrate through the gingivodental junction into the cervical defects. The gingiva around the upper central incisors was pink and firm on probing without bleeding with probing depths of 3 mm (Fig. 3). However, other maxillary and most mandibular teeth showed clinical signs of periodontitis with gingival swelling and positive bleeding after probing. Overhanging fillings of the maxillary and left mandibular premolars and calculus of the maxillary molars were apparently a potential etiologic factor for advanced generalized periodontitis (Fig. 2).

After the examination in our clinic with the diagnosis of multiple idiopathic ECR and Generalized Stage 3 Periodontitis, the patient consulted her endocrinologist, who ordered routine laboratory tests. These test results were (including alkaline phosphatase level) within the normal range with the exception of an elevated cholesterol level of 5.89 mmol/l. In July 2020, circulating intact parathyroid hormone and serum vitamin D, calcium, magnesium and phosphorus levels were normal. Her osteoporosis treatment with denosumab, calcium and vitamin D was assessed as efficient with decreased bone turnover markers: osteocalcin level was 5.06 ng/ml (normal range, 15.0–46.0 ng/ml), procollagen type 1 N-terminal propeptide was 9.27 ng/ml (normal range, 16–76 ng/ml), and collagen type 1 C-terminal telopeptide was 0.051 ng/ml (normal range, 0.104–1.008 ng/ml). Her densitometry in 2010 showed osteoporosis. During a decade of treatment, her bone mineral density increased from 0.528 g/cm^2^ (T-score − 3.4) in 2010 to 0.697 g/cm^2^ (T-score − 2.0) in 2020.

**Fig. 4:**
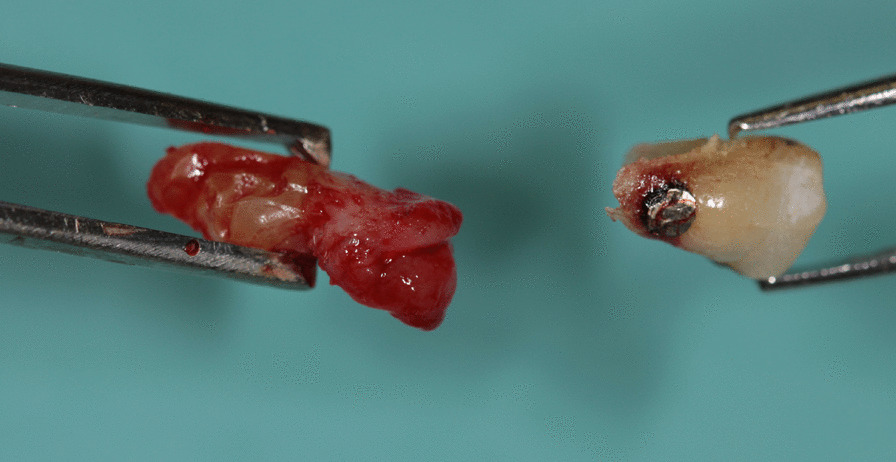
Photograph of extracted tooth 35 showing in detail the resorptive soft tissue process in the cervical area

**Fig. 5:**
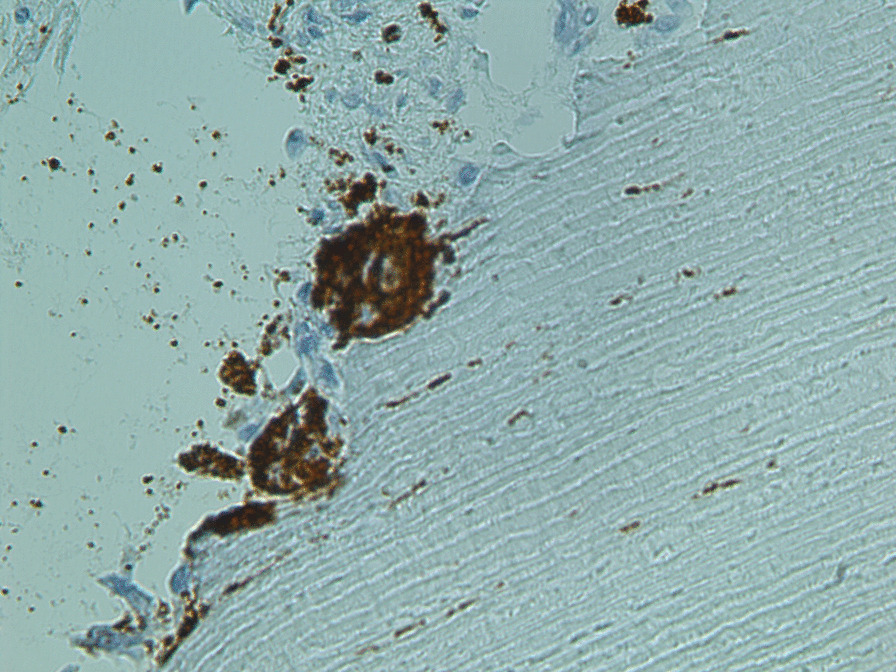
Photomicrograph of biopsy specimens harvested from the resorptive defect of the cervical area of tooth 35. Odontoclasts (CD68+) in detail. Magnification ×480

**Fig. 6:**
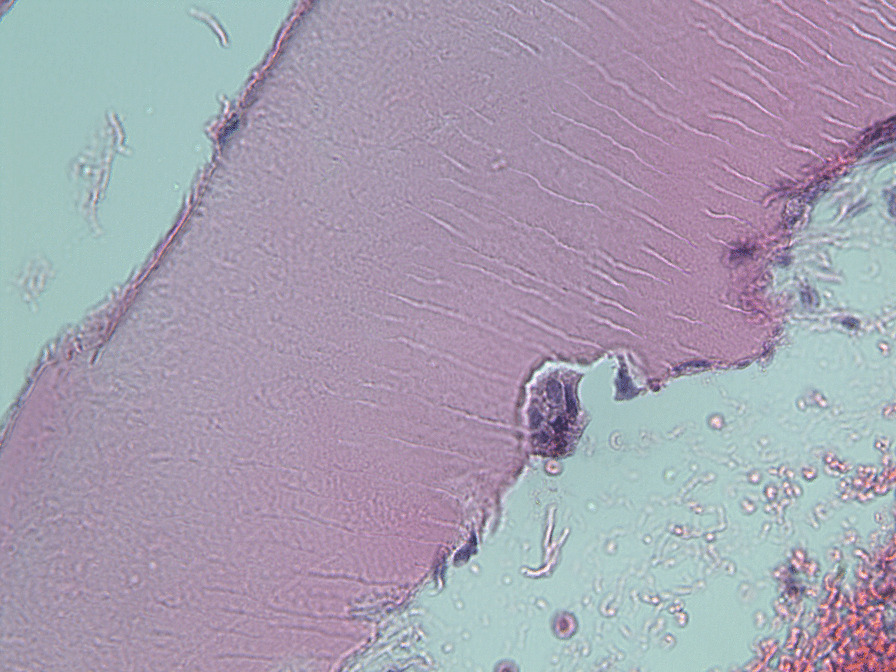
Photomicrograph of biopsy specimens harvested from the resorptive defect of the cervical area of tooth 35. Odontoclast in a resorption lacune in detail. Hematoxylin and eosin stain. Magnification ×240

Because of the pain and mobility of tooth 35, in August 2020, the tooth was extracted under local anesthesia with subsequent suturing of the mucoperiostal flap to avoid medication-related osteonecrosis of the jaw (MRONJ). The extracted root with cervical resorptive lesion was sent for histological examination (Fig. 4). The healing of the wound was uneventful, and stitches were removed after 14 days. Histological examination of the cervical area of the fractured tooth revealed inflammation in connective tissue with the presence of CD68+ (cluster of differentiation) and CD163 + histiocytoid cells as well as CD3+ T lymphocytes and CD20+ B lymphocytes. Sporadically MPO+ (myeloperoxidase positive) leukocytes were present. In surrounding alveolar bone, osteoblasts and sparse osteocytes were found. Connective tissue in areas of resorption contains fibroblast and fibrocytes and osteoclast-like giant cells (CD68+ and CD136+) on the border between dentin and the invading resorptive soft tissue (Figs. 5, 6). Because the other teeth showed no symptoms or mobility, the decision was made to “watch and wait”. The extent of resorptive defects was too large, and the number of teeth involved excluded conservative treatment. In September 2020, the patient lost teeth 22 and 25 due to spontaneous fracture in the cervical area and decided on definitive treatment with extractions of teeth and restoration with removable full dentures.

## Discussion and conclusions

External cervical root resorption is an aggressive pathologic process that may lead to loss of teeth. Multiple ECR of permanent teeth is rare condition. The first case of idiopathic external cervical root resorption was published in 1930 by Mueller and Rony; since then, several cases have been reported in the literature [16–21]. The etiology of ECR remains incompletely characterized. Potential predisposing factors have been identified, such as dental trauma, orthodontic treatment, internal bleaching, periodontal surgery, Paget’s disease of bone, genetic predisposition, cystic lesions, impacted teeth, playing wind instruments, feline viruses transmissed to humans, and osteoclastic rebound effects after cessation of denosumab [3, 20, 22–24]. In the present case, we did not find any of the mentioned etiological factors for ECR, and we observed a progression of ECR despite ongoing antiresorptive therapy with denosumab. Currently, evidence about a possible association between ECR and the use of denosumab is lacking. Since 2013, 31 cases of ECR in patients treated with denosumab for osteoporosis have been recorded (https://www.ehealthme.com/ds/prolia/tooth-resorption/). Some reports in the literature suggest that denosumab or bisphosphonates may act protectively against the aggressive progression of ECR during treatment [20, 21, 25]. The present patient has been treated with denosumab for 9 years without discontinuation of therapy. According to regular monitoring of antiresorptive therapy, her treatment for osteoporosis was effective, exhibiting decreased levels of bone turnover markers and an increase in bone mineral density. Deeb et al. reported a similar case of generalized ECR in a patient previously treated with denosumab for postmenopausal osteoporosis after its discontinuation, suggesting a potential osteoclastic rebound effect as a reason for ECR [20]. Denosumab is a fully human monoclonal antibody with high affinity and specificity for human receptor activator of nuclear factor kappaB ligand (RANKL), which neutralizes the activity of human RANKL, thereby inhibiting osteoclast formation, function and survival [26]. Discontinuation of denosumab therapy has been associated with a significant bone turnover rebound and a rapid loss of bone mass [27]. The rebound effect after cessation of denosumab may lead to a decrease in bone mineral density and an increase in bone turnover markers to above pretreatment baseline levels [11]. Possible explanations for the “rebound effect hypothesis” could be that an increased pool of osteoclast precursors that were dormant during the treatment period with denosumab become activated after its discontinuation and/or that a high RANKL/OPG ratio ensues after denosumab is cleared from the circulation, leading to a rapid rebound in remodeling rates [28]. Because the administration of denosumab in the present case was continuous every six months, the rebound phenomenon was not a presumable cause of progressive ECR. The recent publication by Alyahya et al. suggests that the use of denosumab could significantly predict the risk of developing ECR [29]. Yet the factors that activate osteoclasts/odontoclasts and recruit them to root surfaces rather than bone surfaces (noted in periodontitis) remain unknown [24].

Histological examinations of ECR lesions typically reveal the presence of multinucleated osteoclasts or resorptive (clastic) cells located within resorptive lacunas [30, 31]. In the present case, active osteoclast-like cells were found in the resorptive process, and aggressive progression of root resorption has proceeded during the last 3 years despite ongoing antiresorptive therapy with denosumab. A similar case of multiple idiopathic external root resorptions in a female patient with osteoporosis treated with bisphosphonates was published in 2005 [21]. In that case, ECR was diagnosed before the administration of antiresorptive therapy, no multinucleated osteoclasts were observed on resorpted dentin, and the resorption did not advance for 6 years of treatment with bisphosphonates, suggesting that bisphosphonates may prevent the progression of root resorption [21]. Because denosumab also acts as an inhibitor of osteoclastic formation and activation, a similar prognosis would be expected in the present case. However, in the present case, ECR started and progressed after more than 6 years of antiresorptive therapy with denosumab. This finding suggests that a different mechanism of inducing osteoclastogenesis or activating osteoclastic resorption other than RANK/RANKL signaling must be involved. Osteoclasts and odontoclasts are morphologically and functionally similar multinucleated cells of hematopoietic origin responsible for the resorption of bone or dental hard tissue [32]. RANK and its ligand RANKL have been localized in odontoblasts, pulp fibroblasts, periodontal ligament fibroblasts, and odontoclasts [33]. Osteoclastogenesis is modulated by osteoprotegerin (OPG), a member of the TNF receptor superfamily that inhibits osteoclastogenesis by preventing RANKL from binding to its receptor RANK at the osteoclast membrane. The RANKL/RANK/OPG system is a key mediator in osteoclastogenesis [34]. OPG, RANKL and RANK have also been identified in odontoclasts activated during resorption of deciduous teeth [13, 34]. Recent studies have shown that RANKL/RANK signaling also plays a role in various physiological processes within the immune system [6, 34]. In this case report, we presented an example of multiple ECR in a patient with pre-existing advanced periodontitis. Infection may not be a prerequisite for the initiation of ECR [15]. However, it may lead to damage of the protective cementum layer in the area of cemento-enamel junction, which is one of the known etiological factors for the development of ECR [24]. Periodontitis is known to be associated with elevated levels of pro-inflammatory cytokines such as IL-1, IL-6 and TNF-α. Increased levels of the same inflammatory cytokines have been detected in the gingival crevicular fluid during root resorption [35]. Inflammatory response is indispensable for osteoclastogenesis. IL-1β, IL-6 and TNF-α are recognized as key factors contributing to the upregulation of RANKL expression by studies on both root resorption and periodontitis. Given that the initiation of osteoclastogenesis in root resorption and in periodontitis share a similar mechanism, it is reasonable to infer that resorptive tissues might derive from periodontal tissues to ECR [15].

Interesting in this case report is the coincidence of ongoing RANKL-blocking antiresorptive treatment and massive resorption of all tooth roots. Although RANKL-induced osteoclast formation is considered as major pathway, reports in the literature suggest that osteoclasts can also differentiate independently of RANKL [14, 36–42]. In a sufficiently inflamed environment, other cytokines may compensate to form osteoclast-like cells independently of RANK. O´Brien et al. suggest that TNF/IL-6 can drive RANK-independent osteoclast formation in vivo and in vitro [38]. Feng et al. suggest that several other humoral factors and pro-inflammatory cytokines such as IL-1, IL-6, TNF-α, TGF-β or lipopolysachcaride can substitute for RANKL to induce osteoclast formation [42]. TNF-α has a fundamental role in osteoclastogenesis and may stimulate osteoclast differentiation in the presence of M-CSF independent of the RANK-RANKL system [40, 41]. Macrophage-colony stimulating factor (M-CSF) is an essential factor involved in the proliferation and differentiation of osteoclasts from their progenitors and histological studies show that local trauma to the bone or periodontal ligament increases the concentrations of M-CSF [7–9, 43]. This finding may explain the progressive external root resorption by activated odontoclasts in conditions of suppressed RANK/RANKL signaling during denosumab treatment and advanced periodontitis. Further research is needed to better understand the process of external root resorption and the factors influencing this process in conditions of suppressed RANK/RANKL signaling.

This case report demonstrates aggressive and multiple idiopathic external cervical root resorption that started and advanced during ongoing antiresorptive therapy with the human monoclonal RANKL-blocking antibody denosumab without discontinuation of therapy. This finding suggests that treatment with denosumab may be associated with severe and aggressive odontoclastic resorption of multiple dental roots despite an adequate inhibitory effect on osteoclasts in the treatment of osteoporosis. The RANKL-independent pathways of clastic cell formation are likely to be involved in this pathological process.

## Data Availability

Not applicable.

## Abbreviations

RANK: Receptor activator of nuclear factor kappaB
RANKL: Receptor activator of nuclear factor kappaB ligand
ECR: External cervical root resorption
OPG: Osteoprotegrin
M-CSF: Macrophage-colony stimulating factor
TNF-α: Tumor necrosis factor alpha
IL-1β: Interleukin 1β
MRONJ: Medication-related osteonecrosis of the jaw
CD: Cluster of differentiation
MPO+: Myeloperoxidase positive
TNF: Tumor necrosis factor
IL-6: Interleukin-6
TGF-β: Transforming growth factor beta
